# Gas tamponade followed by laser treatment for macular retinal detachment secondary to optic pit

**DOI:** 10.5935/0004-2749.20230066

**Published:** 2023

**Authors:** Leandro Chaves, Julian Costa, Thaís Bastos, Marina Albuquerque, Ingrid Scott, Rodrigo Jorge

**Affiliations:** 1 Department of Ophthalmology, Otorhinolaryngology and Head and Neck Surgery, Faculdade de Medicina de Ribeirão Preto, Universidade de São Paulo, Ribeirão Preto, SP, Brazil.; 2 Departments of Ophthalmology and Public Health Sciences, Penn State College of Medicine, Hershey, Pennsylvania, USA.

**Keywords:** Optic disk/abnormalities, Optic nerve diseases/complications, Retinal detachment, Laser therapy, Intravitreal injections, Fluorocarbons/administration & dosage, Gases/administration & dosage, Disco óptico/anormalidades, Doenças do nervo óptico/complicações, Descolamento retiniano, Terapia a laser, Injeções intravítreas, Fluorcarbonetos/administração & dosagem, Gases/administração & dosagem

## Abstract

**Purpose:**

The study aimed to describe anatomic and visual outcomes associated with perfluoropropane intravitreal injection followed by laser treatment for macular retinal detachment secondary to optic disc pit.

**Methods:**

A single-center, retrospective study. Medical records of all patients treated at a tertiary retina referral center were evaluated between 2011 and 2018 for congenital optic disc pit-associated macular detachment with 0.3 ml 100% perfluoropropane intravitreal injection followed by retinal laser photocoagulation along the temporal optic disc margin as the initial treatment.

**Results:**

Six patients with optic disc pit-associated macular detachment were identified, with postoperative follow-up ranging from 13 to 52 months (mean: 28 months). Spectral domain optical coherence tomography (SD-OCT) showed complete fluid resolution without recurrence in five of the six cases. Four cases showed complete reabsorption after Intravitreal perfluoropropane plus laser, one patient needed an extra procedure (pars plana vitrectomy with inner limiting membrane peeling and pedicle flap inversion over the temporal optic disc margin) to achieve complete fluid reabsorption, and one patient had persistent intraretinal fluid and denied additional surgeries. The time between the initial procedure and total fluid reabsorption varied from 6.5 to 41 months (mean: 19.5 months). Best-corrected visual acuity improved after surgery on the last follow-up visit in all cases.

**Conclusion:**

100% perfluoropropane intravitreal injection followed by photocoagulation along temporal optic disc margin was associated with anatomic and visual improvement in most cases, representing an alternative treatment approach for optic disc pit-associated macular detachment.

## Abstract (pt)

**Objetivo:**

Descrever os resultados anatômicos e visuais associados à injeção intravítrea de perfluoropropano seguida de tratamento a laser para descolamento de retina macular secundário à fosseta do disco óptico.

**Métodos:**

Estudo retrospectivo em um único centro. Foram revisados os prontuários médicos dos pacientes com descolamento macular associado a fosseta do disco óptico congênito em um centro de referência terciário de retina entre 2011 e 2018. Todos receberam como estratégia de tratamento inicial injeção intravítrea de perfluoropropano 100% seguido por fotocoagulação a laser ao longo da margem temporal do disco óptico.

**Resultados:**

Foram identificados seis pacientes com descolamento macular associado a fosseta do disco óptico durante o período do estudo. O seguimento pós-operatório variou de 13 a 52 meses, com média de 28 meses. SD-OCT demonstrou resolução completa do fluido em cindo dos seis casos, sem recorrência. Quatro casos apresentaram reabsorção completa após perfluoropropano intravítreo associado a laser, e um paciente necessitou de procedimento adicional (vitrectomia via *pars plana* com *peeling* da membrana limitante interna e inversão do retalho do pedículo sobre a margem temporal do disco óptico) para obter reabsorção completa de fluidos. Um paciente apresentou fluido intrarretiniano persistente e negou tratamentos adicionais. O tempo entre o procedimento inicial e a resolução completa do fluido variou entre 6,5 a 41 meses, com média de 19,5 meses. A acuidade visual corrigida melhorou após a cirurgia, considerando a última consulta de acompanhamento em todos os casos.

**Conclusão:**

A injeção intravítrea de perfluoropropano 100% seguida de fotocoagulação ao longo da margem temporal da margem do disco óptico foi associada à melhora anatômica e visual na maioria dos casos e representa uma abordagem terapêutica alternativa para o descolamento macular associado a fosseta do disco óptico.

## INTRODUCTION

Optic disc pit was first reported in 1882 by Wiethe^(1)^. Optic disc pits (ODP) are a part of cavitary optic disc anomalies set that includes coloboma, morning glory disc, and extrapapillary cavitation^(2)^, characterized by abnormal communication between the intraocular and extraocular spaces. ODPs are extremely rare, without gender predilection, and can be congenital or acquired. The estimated congenital form incidence is 1:11,000 persons. Only 15% of these patients have a bilateral disease, and, in most cases, there is only one pit per disc, with a typical inferotemporal pit location^(3)^. The pits are oval-shaped depressions that are frequently gray but may be yellowish or black^(2,3)^.

Macular involvement was first described in 1927 by Halbertsma^(4)^. ODPs are typically asymptomatic, although as many as 75% of affected patients develop retinal detachment or macular retinoschisis, experiencing an impaired vision, an advanced condition called optic disc pit maculopathy^(3,5)^.

ODP-associated retinal detachment pathophysiology and subretinal fluid etiology are controversial. Two studies have proposed a mechanism of subretinal fluid accumulation, such as its origin from the vitreous cavity and subarachnoid space^(2,3)^. Subretinal fluid biochemical analysis in two patients demonstrated a composition similar to that of cerebrospinal fluid^(6)^. In contrast to the theory proposed by Lincoff et al.^(7)^, which describes retinal schisis presence preceding subretinal fluid accumulation, optic coherence tomography (OCT) studies have demonstrated that retinal schisis does not always exist in patients with ODP-associated retinal detachment^(8)^.

The prognosis of untreated ODP-associated macular detachment varies. Brown et al.^(9)^ followed 20 eyes with untreated optic disc maculopathy for more than one year. The authors showed subretinal fluid persistence in 75% of these cases and last follow-up visual acuity of 20/100 or worse in 55% of cases. Sobol et al.^(10)^ followed 15 eyes with ODP-associated with macular detachment for 9 years and showed that 80% of them had a visual acuity of 20/200 or worse at the last follow-up.

There is no universally accepted effective therapy for ODP-associated macular detachment. Various treat­ments have been described, including intraocular gas injection only^(11)^, gas injection with laser photocoagulation along the temporal optic disc margin^(12)^, macular buckling^(13)^, pars plana vitrectomy with gas tamponade with or without laser, pars plana vitrectomy with internal limiting membrane peeling with or without a flap^(14)^, platelet-rich autologous serum infusion^(14,15)^, autologous scleral plug^(16)^, and partial inner retinal fenestration^(17)^.

Herein, we report the results of a minimally invasive and low-cost treatment with a combination of 100% perfluoropropane (C3F8) intravitreal injection followed by retinal laser photocoagulation along the temporal optic disc margin as the initial treatment for ODP-associated macular detachment.

## METHODS

This study adhered to the tenets of Declaration of Helsinki and was approved by the Research Ethics Committee of Ribeirão Preto School of Medicine - University of São Paulo (CAAE: 32397720.0.0000.5440).

Medical records of patients that were treated at the *Hospital das Clínicas da Faculdade de Medicina de Ribeirão Preto - Universidade de São Paulo* between 2011 and 2018 for congenital ODP-associated macular detachment with 100% C3F8 intravitreal injection followed by retinal laser photocoagulation along the the temporal optic disc margin as the initial treatment for their ODP-associated macular detachment were reviewed.

Collected data included age at diagnosis, gender, laterality, duration of symptoms, visual acuity on presentation, retinal layers affected on OCT scans, need for retreatment or alternative therapy, visual acuity at last follow-up, the time course of visual acuity improvement, and follow-up duration. The initial treatment was provided by the same surgeon in an ambulatory surgical environment under aseptic conditions and with topical anesthesia. After paracentesis of the anterior chamber, 0.3 ml 100% C3F8 was injected intravitreally, and the patient was instructed to maintain face-down positioning for seven days. Retinal photocoagulation with green diode laser was performed along the temporal optic disc margin three days after C3F8 injection using the following parameters: 50-100 µm spot size with an exposure duration of 50-100 milliseconds and sufficient intensity to achieve visible marks. All patients were treated with 360-degree peripheral retinal laser photocoagulation before C3F8 injection for a theorical retinal detachment prophylaxis.

Green diode laser retinal photocoagulation along the temporal optic disc rim was repeated in five patients 30-70 days after the first procedure. The laser was repea­ted once the surgeon observed no improvement of the intraretinal and/or subretinal fluid on OCT follow-up. The mean number of laser sessions was 2.2, with a mean number of spots being 53. The laser was repeated at the same spot when the laser burn performed in a previous section was not clearly visible or in an additional row temporal to the previous section to stimulate a healthier retinal pigment epithelium (RPE) to generate a more efficacious chorioretinal adhesion than the previous one.

## RESULTS

Six patients with ODP-associated macular detachment during the study period were identified (Table 1), and all these ODP were congenital. Three patients were males, and one had bilateral involvement but consented to treatment only for his left eye. The age at diagnosis ranged from 5 to 52 years, with a mean age of 24.3 years (standard deviation (SD): 20.76). Duration of symptoms ranged from eight days to five years. Mean best-corrected visual acuity (BCVA) at four meters was 20/200 or worse in three cases and 20/40 or better in two.

**Table 1:** Clinical characteristics of 6 patients with optic disc pit maculopathy, OCT finding, and outcomes

Patient # /gender/age (year)/eye	Symptons duration (month)	Fluid locaction	Retinal layers breaks	Baseline visual acuity	Final visual acuity	LASER section
1 / M / 10 / RE	10	SRF	NO	20/40	20/20	2
2 / F / 14 / RE	60	SRF + ONL	OPL+ ONL + ELM + IS/OS + ZI	20/2.000**	20/25	2
3* / M / 5,0 / LE	24	NFL + GCL + INL + ONL	NO	20/80	20/32	3
4 / M / 12 / LE	0,26	SRF + NFL + GCL + INL + ONL	OPL+ ONL + ELM + IS/OS + ZI	20/200	20/25	2
5 / F / 52 / LE	1	SRF + GCL + ONL + INL	OPL+ ONL + ELM + IS/OS + ZI	20/2.000**	20/200	3
6 / F / 8 / RE	1	SRF	IS/OS + IZ	20/30	20/20	1

* Patient with pit in both eyes.

M= male; F= female= RE= right eye; LE= left eye; SRF= subretinal fluid; ONL= outer nuclear layer; GCL= ganglion cell layer; INL= inner nuclear layer; NFL= nerve fiber layer; PPV= *pars plana vitrectomy*; ILM= internal limiting membrane, OPL: outer plexiform layer; ELM= external limiting membrane; IS/OS= inner segment outer segment junction layer; IZ= interdigitation zone

** Visual acuity conversion reference^(21)^.

**Table 1:** Clinical characteristics of 6 patients with optic disc pit maculopathy, OCT finding, and outcomes.

LASER spots number	Results	Aditional aproach	Carbonic anhydrase inhibitor use	Follow-up (month)
30	Total fluid reabsorption	NO	NO	12
81	Total fluid reabsorption	NO	YES, eye drops	19
38	Partial reabsorption	NO	YES, sistemic	25
36	Total fluid reabsorption	NO	YES, sistemic	52
80	Total fluid reabsorption	PPV + ILM peeling	YES, sistemic	47
53	Total fluid reabsorption	NO	NO	13

Spectral domain optical coherence tomography (SD-OCT) (Spectralis OCT^®^, Heidelberg Engineering, Heidelberg, Germany) revealed optic disc defect and macular subretinal fluid in all patients. Four patients had intraretinal fluid involving primarily the outer nuclear layer. In three cases, there was fluid in the inner and outer retina. Retinal layer discontinuity was observed in four eyes, all of them involving the inner segment/outer segment (IS/OS) layer, interdigitation zone, and RPE. Involvement of the outer plexiform and outer nuclear layers and the external limiting membrane was observed in three cases (Table 1).

**Figure 1:**
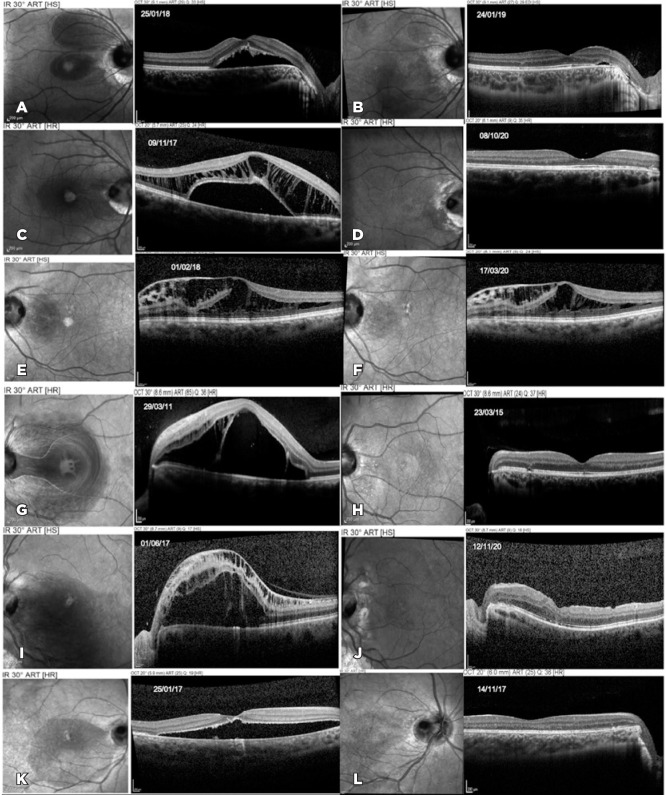
Patients number 1-6: Optical coherence tomography (OCT) before and after treatment. OCT images showing improvement of macular fluid in all six patients. All patients were treated with 0.3 ml 100% C3F8 intravitreal injection followed by retinal photocoagulation along the temporal optic disc margin. Patient 1 (A, B); 2 (C, D), 4 (G, H), and 6 (K, L) showed anatomic improvement and complete resolution of macular fluid after this treatment. Patient 5 (I, J) had fluid recurrence and needed an additional procedure (inner limiting membrane peeling under brilliant blue dye visualization, pedicle flap inversion at the temporal optic disc margin). There was complete resolution of the fluid after this treatment. Patient 3 (E, F) showed a slight improvement of the intraretinal fluid. However, he refused additional treatments and the fluid persisted.

Postoperatively, OCT demonstrated complete fluid resolution in five patients, without recurrence during follow-up (Figure 1). Total reabsorption was documented after 12 months, 13 months, 25 months, and 6.5 months in patients number 1, 2, 4, and 6, respectively. Patient number 5 failed to demonstrate fluid reabsorption on OCT and underwent pars plana vitrectomy, achieving complete fluid resolution 41 months after the initial treatment. Patient number 3 failed to demonstrate progressive fluid reabsorption and refused any additional treatment.

Patient number 5 demonstrated transient anatomical improvement but had subretinal/intraretinal fluid recurrence and underwent 25-gauge pars plana vitrectomy, inner limiting membrane peeling under visualization with brilliant blue dye, inversion of pedicle flap at the temporal optic disc margin, and 15% C3F8 intravitreal injection 5 months after the initial procedure (gas injection followed by photocoagulation).

Four patients received carbonic anhydrase inhibitors during the postoperative period: orally in three cases, (250 mg-1 g per day) and topically (every 8 hours) in the fourth case. The duration of medication use ranged from 30 days to 30 months.

BCVA improved, at least in one line, in all patients. Median BCVA was 20/80 before surgery and improved significantly to 20/32 at last follow-up after intravitreal gas plus laser. Four patients had BCVA of 20/40 or better. The median follow-up duration was 28 months (range: 12-52 months).

## DISCUSSION

Using laser alone to treat OPD-associated macular detachment was first reported in 1969 by Gass^(18)^, who used xenon laser to create chorioretinal adhesion at the temporal optic disc margin in an attempt to minimize fluid movement from the pit to the subretinal space in two patients. In 1972, Mustonen and Varonen used argon laser in three patients with the same objective and observed slow subretinal fluid reabsorption^(19)^. Sandali et al. reported a success rate of 30% using laser alone^(20)^. The use of gas was evaluated by Lincoff et al. only in 1998 in three cases. Despite an initial visual acuity improvement, macular fluid recurrence was seen in two cases within 3 months and in the third case during the fifth year of follow-up^(21)^. In our patients, we used intravitreal C3F8 in addition to the laser, in a similar way to previously published studies^(12,20,22-24)^. With proper positioning, the gas bubble exerts pressure on the inner retinal surface and induces passive subretinal fluid migration through the RPE and choroid. Consequently, it reduces the distance between the retinal layers, thus, facilitating photocoagulation and subsequent adhesion between the outer retinal layers and RPE^(2,12,22)^.

Our patients and results are comparable to the published data. Two-thirds of our patients were younger than 14 years, and the mean age of 24.3 years was comparable to Lei et al. (25.8 years) and Elmohamady et al. (22 years)^(22,24)^. Our success rate was 88.3%, which is comparable to previous studies as well, where Sandali et al. reported a success rate of 72%^(20)^; Lei et al. reported a success rate of 75%^(22)^, and Elmohamady et al. reported a success rate of 82%^(24)^.

The duration between C3F8 injection and retinal photocoagulation along the temporal optic disc margin in our patients was three days, in contrast to the study by Lei et al.^(22)^, who applied laser one or two weeks after gas injection, and the study by Elmohamady et al.^(24)^, who applied laser two or three weeks after gas injection. We prefer a shorter period of face-down positioning, which is more convenient for the patient. Another difference in our technique compared to that of Lei et al.^(22)^ was the C3F8 concentration, which was 100% in the present study and 66% in the former. We prefer a higher C3F8 concentration since that usually generates a larger bubble exerting a higher pressure over a greater retinal area; this, in turn, may be associated with more efficient intraretinal and subretinal fluid drainage. Elmohamady et al. had used 0.6 ml of 100% sulfur hexafluoride (SF_6_)^(24)^.

All patients were treated with 360-degree peripheral retinal laser photocoagulation prior to intravitreal C3F8 injection for rhegmatogenous retinal detachment prophylaxis. Although ODP is not related to a higher risk of rhegmatogenous retinal detachment or peripheral retinal abnormalities, we believe that posterior hyaloid detachment, expansion, movements, and mechanical forces triggered by the intraocular gas may generate traction on the peripheral retina, with the risk of retinal tear and detachment, as reported by the DRCR Retina Network^(25)^, where intravitreal perfluoropropane was used for vitreomacular traction with and without macular hole. The prophylaxis might not have been necessary since other similar case series that used gas injection without pars plana vitrectomy to treat optic disc pit maculopathy did not describe rhegmatogenous retinal detachment nor peripheral breaks during the follow-up^(12,20,22,24)^. Fortunately, none of these complications were identified during the follow-up in our study, and no patient developed epiretinal membrane.

An important characteristic of the treatment strategy reported in this study is slow intra- and subretinal fluid resolution. The fastest total reabsorption was observed 6.5 months after the treatment. Visual acuity started to improve concomitantly with the beginning of fluid reabsorption identified on OCT. We believe that careful follow-up of patients who are improving is preferable to administering multiple treatments. In contrast, visual acuity worsening associated with increased intraretinal or subretinal fluid on OCT is an indication for additional treatment, as was the case for patient number 5 (who was treated with pars plana vitrectomy).

Visual acuity improved in all patients in our study. There was no significant association between discontinuity of the retinal layers on OCT or duration of symptoms and visual prognosis.

Progressive fluid reabsorption and the closure of communication between the subretinal and intraretinal spaces with the optic pit occurred in 5 of the 6 cases. Four of them showed anatomic improvement after intravitreal gas injection, face-down positioning, and laser photocoagulation. One of them needed an additional procedure (pars plana vitrectomy, inner limiting membrane peeling under brilliant blue dye visualization, and pedicled flap inversion at the temporal optic disc margin). This patient was the oldest from the study (a 52-year-old woman) and probably had greater vitreous gel liquefaction. We propose two potential explanations for first treatment failure. 1) Even with face-down positioning, there is not enough time for retinal compaction; therefore, the laser may not have a healing effect on all retinal layers. The liquefied vitreous gel may infiltrate the intra- and subretinal space more easily as soon as the patient abandons face-down positioning even for short time intervals. 2) A lower healing capacity of older patients. For this reason, pars plana vitrectomy may be necessary in older patients. During vitrectomy, inner limiting membrane peeling is performed, and a pedicle internal limiting membrane flap is prepared and positioned over the pit in an attempt to prevent fluid entry from the vitreous body into the sub- or intraretinal space. Further, vitrectomy permits the placement of a more voluminous C3F8 bubble, which probably leads to a more effective and long-lasting tamponade of the temporal optic disc margin. This provides a longer healing time after retinal photocoagulation, which may be performed intra- or postoperatively. Some patients with ODP-associated macular detachment are treated only by vitrectomy, without using flaps or endolaser. Those who recommend this technique believe that removal of the hyaloid and prediscal membranes may create a communication between the subarachnoid space and the vitreous cavity, preventing the fluid from that space from migrating toward the retinal tissue.

Carbonic anhydrase inhibitor use varied among patients according to an individualized approach and respecting the tolerance and contraindications for each case. The rationale for this prescription is stimulating the mechanism of fluid pumping outside the subretinal space by RPE cells. Although literature data suggest a theoretical benefit of the carbonic anhydrase inhibitor use in this condition^(26-28)^, the current small case series do not permit an assessment of the potential carbonic anhydrase inhibitors impact on OPD-associated macular detachment.

Clinical examination of patient number 4 showed a possible optic disc pit in the right eye (contralateral eye), which was confirmed on OCT (Figure 1). Cases of optic disc pit not associated with maculopathy should not be treated prophylactically with laser along the temporal optic disc margin. The natural history of this disease may not involve maculopathy development, and the risk of injury to the papillomacular bundle does not justify this approach^(2)^.

The present study is limited by the small sample size, its retrospective nature, and lack of a control group. The postoperative treatment was not uniform, and two-thirds of the patients received anhydrase carbonic medications by different routes and during different periods. Low ODP-associated maculopathy incidence and multiple therapeutic options existence represent challenges in conducting comparative studies.

The procedure described herein represents a low-cost alternative therapeutic approach to manage ODP-asso­ciated macular detachment and is associated with outcomes similar to those obtained with the surgical procedures mentioned earlier^(20)^. It may be of particular interest in countries with limited public health resources and a lack of universal access to vitreoretinal surgeries.
